# Complement Inhibition Targeted to Injury Specific Neoepitopes Attenuates Atherogenesis in Mice

**DOI:** 10.3389/fcvm.2021.731315

**Published:** 2021-09-28

**Authors:** Shen Dai, Fengming Liu, Mi Ren, Zhongnan Qin, Namita Rout, Xiao-Feng Yang, Hong Wang, Stephen Tomlinson, Xuebin Qin

**Affiliations:** ^1^Division of Comparative Pathology, Tulane National Primate Research Center, Covington, LA, United States; ^2^Department of Microbiology and Immunology, Tulane University School of Medicine, New Orleans, LA, United States; ^3^Department of Neuroscience, Temple University Lewis Katz School of Medicine, Philadelphia, PA, United States; ^4^Division of Microbiology, Tulane National Primate Research Center, Covington, LA, United States; ^5^Center for Metabolic Disease Research and Cardiovascular Research, Temple University Lewis Katz School of Medicine, Philadelphia, PA, United States; ^6^Department of Microbiology and Immunology, Medical University of South Carolina, Charleston, SC, United States

**Keywords:** atherosclerosis, oxidization, natural antibody, animal–mouse, complement

## Abstract

**Rationale:** Previous studies have indicated an important role for complement in atherosclerosis, a lipid-driven chronic inflammatory disease associated to oxidative stress in the vessel wall. However, it remains unclear how complement is activated in the process of atherogenesis. An accepted general model for complement activation in the context of ischemia reperfusion injury is that ischemia induces the exposure of neoepitopes that are recognized by natural self-reactive IgM antibodies, and that in turn activate complement.

**Objective:** We investigated whether a similar phenomenon may be involved in the pathogenesis of atherosclerosis, and whether interfering with this activation event, together with inhibition of subsequent amplification of the cascade at the C3 activation step, can provide protection against atherogenesis.

**Methods and Results:** We utilized C2scFv-Crry, a novel construct consisting of a single chain antibody (scFv) linked to Crry, a complement inhibitor that functions at C3 activation. The scFv moiety was derived from C2 IgM mAb that specifically recognizes phospholipid neoepitopes known to be expressed after ischemia. C2scFv-Crry targeted to the atherosclerotic plaque of *Apoe*^−/−^ mice, demonstrating expression of the C2 neoepitope. C2scFv-Crry administered twice per week significantly attenuated atherosclerotic plaque in the aorta and aortic root of *Apoe*^−/−^ mice fed with a high-fat diet (HFD) for either 2 or 4 months, and treatment reduced C3 deposition and membrane attack complex formation as compared to vehicle treated mice. C2scFv-Crry also inhibited the uptake of oxidized low-density-lipoprotein (oxLDL) by peritoneal macrophages, which has been shown to play a role in pathogenesis, and C2scFv-Crry-treated mice had decreased lipid content in the lesion with reduced oxLDL levels in serum compared to vehicle-treated mice. Furthermore, C2scFv-Crry reduced the deposition of endogenous total IgM in the plaque, although it did not alter serum IgM levels, further indicating a role for natural IgM in initiating complement activation.

**Conclusion:** Neoepitope targeted complement inhibitors represent a novel therapeutic approach for atherosclerosis.

## Introduction

Clinical histological studies indicate that the complement system plays a critical role in atherogenesis (1–3). Recently, we (4) and others (5–7) suggested an atherogenic role for the membrane attack complex (MAC), the terminal product of complement activation, and an anti-atherogenic role for CD59, a MAC inhibitor. The complement system is usually activated via one of three pathways (classical, alternative and lectin pathways) which converge at C3 cleavage, leading to the formation of C3 and C5 convertases, and finally assembly of the MAC (8, 9). The MAC is a cytolytic macromolecular pore that can insert into host cell membranes in pathological conditions (8, 10, 11). Of note, since self-nucleated cells express various complement regulators and have intrinsic anti-lytic mechanisms (12), the MAC may mediate sub-lytic effects in the cells in disease conditions. The sub-lytic MAC formed on cell membranes may activate signaling cascades (13–21), leading to an inflammatory response (22–24). More than 10 plasma- and membrane-bound inhibitory proteins have been identified that restrict complement activation at different stages of the cascade and protect self cells from MAC attack (8, 9). CD59 is the most important membrane inhibitor for specifically restricting MAC formation (25). Previously, we showed that overexpression of human CD59 (hCD59) or inhibition of MAC formation protected against atherogenesis in mice (4, 26). Deficiency of C6, a component necessary for MAC formation, attenuated atherogenesis (6, 27–29). We have also shown that CR2-Crry, an inhibitor of all complement pathways at the C3 activation step and which is targeted to sites of C activation, protects *Apoe*^−/−^ mice against the development of atherosclerosis (26). Recent human studies indicate that some complement components or complement activation products are associated with the occurrence and development of coronary heart disease (30) and subclinical atherosclerosis in systemic lupus erythematosus patients (31). These clinical results further support the critical atherogenic role of the complement system. However, it remains unclear how complement is activated in the process of atherogenesis, and whether inhibition of earlier complement activation products together with the MAC would provide optimum protection against atherogenesis. In this context, it has been shown that C5a inhibition also reduces atherogenesis, whereas C5a supplementation accelerates it (32, 33).

Atherosclerosis is a lipid-driven chronic inflammatory disease. Accumulation of oxidized low-density lipoprotein (oxLDL) in vessel walls is the central event in the initiation and progression of atherosclerotic plaque (34). OxLDL results in the formation of neoepitopes from lipid peroxidation and contributes to plaque development via several mechanisms, including endothelial dysfunction, foam cell formation, and activation of an inflammatory response (35, 36). Previous studies also suggest oxLDL plays a role in complement activation. In *Apoe*^−/−^ mice, immune complexes of oxidized lipids and immunoglobulins (Igs) activate the classical complement pathway, leading to leukocyte infiltration of atherosclerotic lesions (37). *In vitro* studies also suggest that human oxLDL-IgG immune complexes activate complement via the classical pathway (38). Meanwhile, cholesterol crystals derived from oxLDL (39) present in early atherosclerotic plaque (40) and induce complement activation via both the lectin and classical pathways, which may represent the initial complement activation trigger in the plaque (41). Natural self-reactive IgM antibodies present in plaque is another potential complement activator. It has been demonstrated that natural IgM antibodies recognizing injury-induced phospholipid neoepitopes activate complement in an ischemia reperfusion injury model (42). However, whether a similar phenomenon exist in atherosclerosis is not clear.

To examine these questions, we utilized a novel complement inhibitor that is specifically targeted to injury induced phospholipid neoepitopes, in atherosclerosis mouse models. The inhibitor, C2scFv-Crry, consists of single-chain variable fragment (scFv) derived from a natural IgM mAb (C2) linked to Crry, a murine inhibitor of C3 activation (43). C2scFv-Crry recognizes a subset of phospholipids exposed on injured cells post-ischemia injury (42), and it has been demonstrated that C2scFv-Crry is protective in arthritis (44) and lung transplantation (45) mouse models. Since C2scFv specifically recognizes phospholipids, which are components of oxLDL (46), we sought to determine the targeting and therapeutic effect of C2scFv-Crry in a model of atherosclerosis. We found that C2scFv-Crry treatment significantly attenuates atherosclerosis by targeting the plaque. which is associated with decreased complement activation, lipid and IgM deposition.

## Methods

### Production and Purification of C2scFv-Crry

C2scFv-Crry was constructed and purified as previously described (45). The purity of C2scFv-Crry was determined by SDS-PAGE and complement inhibitory activity confirmed by zymosan assay.

### Animal Treatment and Characterization of Atherosclerotic Lesions

*Apoe*^−/−^ mice (JAX 002052) were purchased from The Jackson Laboratory (Bar Harbor, ME) and housed in Lewis Katz School of Medicine at Temple University and Tulane University School of Medicine. To investigate the effect of C2scFv-Crry in the development of atherosclerosis, 8-week-old male *Apoe*^−/−^ mice were treated with C2scFv-Crry (intravenously, 0.25 mg/mouse, twice per week) or the same volume of PBS (intravenously, twice per week) and maintained on a high-fat diet (HFD) (C12108; Research Diets, Inc.) for 2 or 4 months. At the end of the experiment, mice were euthanized by CO_2_ asphyxiation. Serum was collected and the atherosclerotic lesion was characterized as previously described (26, 47). Briefly, the entire aorta from the heart outlet to iliac bifurcation was collected and stained with Oil Red O. The percentage of plaque area (%) was calculated as (Oil Red O-stained area/total aorta area) × 100. The aortic root was snap frozen in optimal cutting temperature (OCT) compound and sectioned (5 μm). Slides were stained with either hematoxylin and eosin (H&E) or Oil Red O, and the plaque area and lipid deposition were calculated. To determine the target deposition of C2scFv-Crry in the plaque, sections of aortic root from PBS or C2scFv-Crry-treated mice were stained with either Alexa Fluor-488 (AF488) isotype antibody or AF488 anti-His antibody (Invitrogen, 4E3D10H2/E3). All animal experiments were reviewed and approved prior to commencement of activity by the Institutional Animal Care and Use Committee at Temple University.

### Immunofluorescence and Immunohistochemistry Staining

Frozen sections of aortic root (5 μm) were stained with 1) rat anti-mouse C3, IgG2a (clone: 3/26, Hycult Biotechnology), which recognizes mouse complement protein C3 as well as activated C3 fragments C3b, iC3b, and C3c; 2) rabbit anti-rat C9, which cross-reacts with mouse C9 (kindly provided by Dr. P. Morgan, University of Wales); 3) rat anti-mouse CD68, IgG2a (clone: FA-1, AbD Serotec) for mononuclear phagocytes; 4) goat anti-mouse IgM (Bio-rad); and 5) rabbit anti alpha smooth muscle actin (SMA-α) (Abcam). All primary antibodies were detected using corresponding secondary antibodies and compared with negative controls, which were stained with the secondary antibody alone. For IgG staining, the sections were directly stained with Alexa Flour 594-conjugated anti-mouse IgG antibody (Invitrogen). For TUNEL staining, the sections were stained with in situ cell death detection kit, Fluorescein (Roche) according to the manufacture's instruction. We quantified immunofluorescence and histological results from three serial sections from each mouse using Image ProPlus 6.0 software as described previously (48). The means of the quantitative results of three sections obtained from each mouse were used to perform the statistical analysis.

### Serum Lipid and oxLDL Measurement

Serum cholesterol and triglyceride profiles were measured at NIH/NIAAA as previously described (49). Serum oxLDL level was determined by oxLDL ELISA kit (Mybiosource) according to the manufacturer's instructions.

### OxLDL Uptake Assay

Mouse primary peritoneal macrophages were isolated at 3 days after i.p. injection of 3% thioglycollate broth medium as previously described (50). For oxLDL uptake assay, 50 μg/ml Dil-labelled oxidized LDL (Dil-oxLDL) (Thermo Fisher) was pre-incubated with PBS or 20 μg/ml C2scFv-Crry for 30 min, followed by incubation with peritoneal macrophages for 16 h. The uptake of Dil-oxLDL was determined by the intensity of Dil (PE channel) in macrophages measured by flow cytometry analysis. In some experiments, macrophages were treated with PBS, oxLDL+PBS, oxLDL+C2scFv-Crry for 24 h and RNA was extracted. The mRNA level of IL-1β was determined by qRT-PCR as previously described (50).

### Statistical Analysis

Experimental results are shown as the mean ± SEM. The difference between the two groups was examined with a nonparametric Mann-Whitney test. All statistical results with p < 0.05 were considered significant.

## Results

### C2scFv-Crry Targets Atherosclerotic Plaque

**Figure 1:**
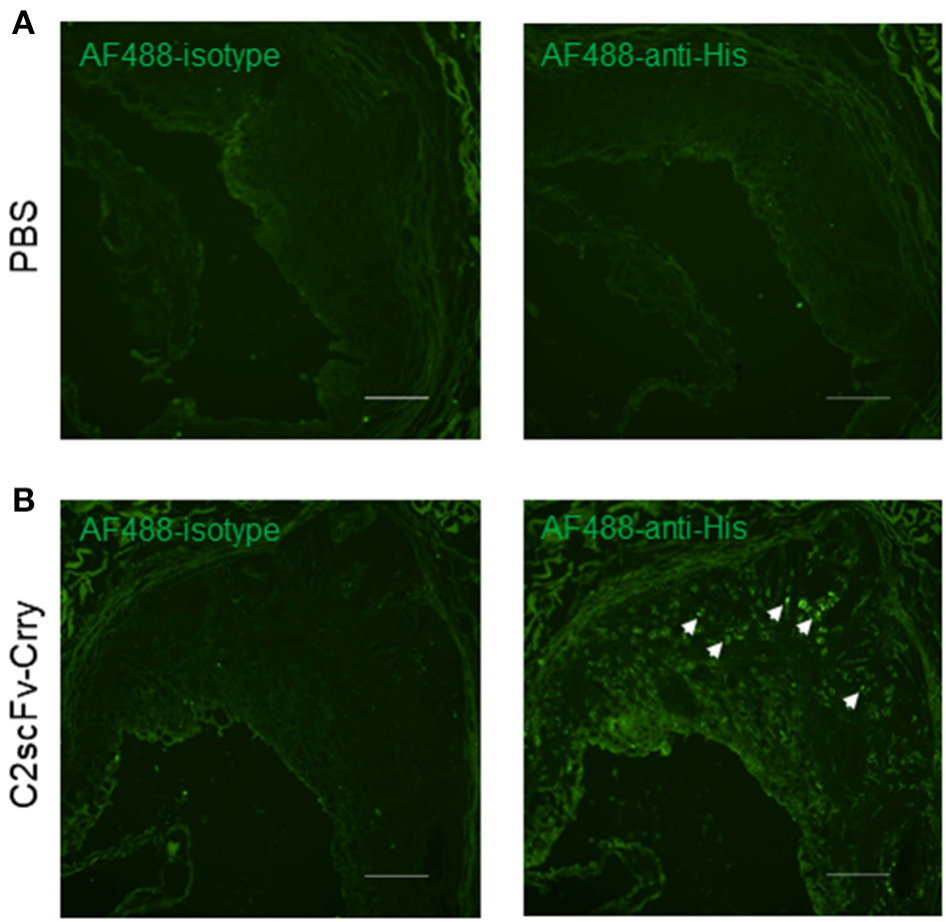
Deposition of C2scFv-Crry in the atherosclerotic plaque of Apoe^−/−^ mice. Aortic root cryosections from Apoe^−/−^ mice treated with PBS **(A)** or C2scFv-Crry **(B)** were stained with Alexa Fluor 488 (AF488)-Isotype (left) or anti-His antibody (right). Representative image shows the deposition in the plaque of C2scFv-Crry-treated mice. Scale bar 100 um.

To determine whether C2scFv-Crry specifically localizes to the atherosclerotic plaque in a therapeutic paradigm, we administered C2scFv-Crry (0.25 mg/mouse) or PBS to 2-month-old *Apoe*^−/−^ mice twice per week for 2 months and maintained the mice on a high-fat diet (HFD). We then immunostained aortic root sections with anti-His antibody, which recognizes the 6xHis tag conjugated to the N-terminus of C2scFv-Crry. Immunofluorescent (IF) staining showed extensive deposition of the His tag in aortic root of C2scFv-Crry-treated mice, but not in PBS-treated mice (Figures 1A,B, right), Of note, there was no positive staining in mice with an isotype control antibody (Figures 1A,B, left). Thus, the C2 neoepitope is expressed in atherosclerosis plaque, which facilitates the targeting of C2scFv-Crry to the plaque.

### C2scFv-Crry Treatment Attenuates the Development of Atherosclerosis in *Apoe*^–/–^ Mice

**Figure 2:**
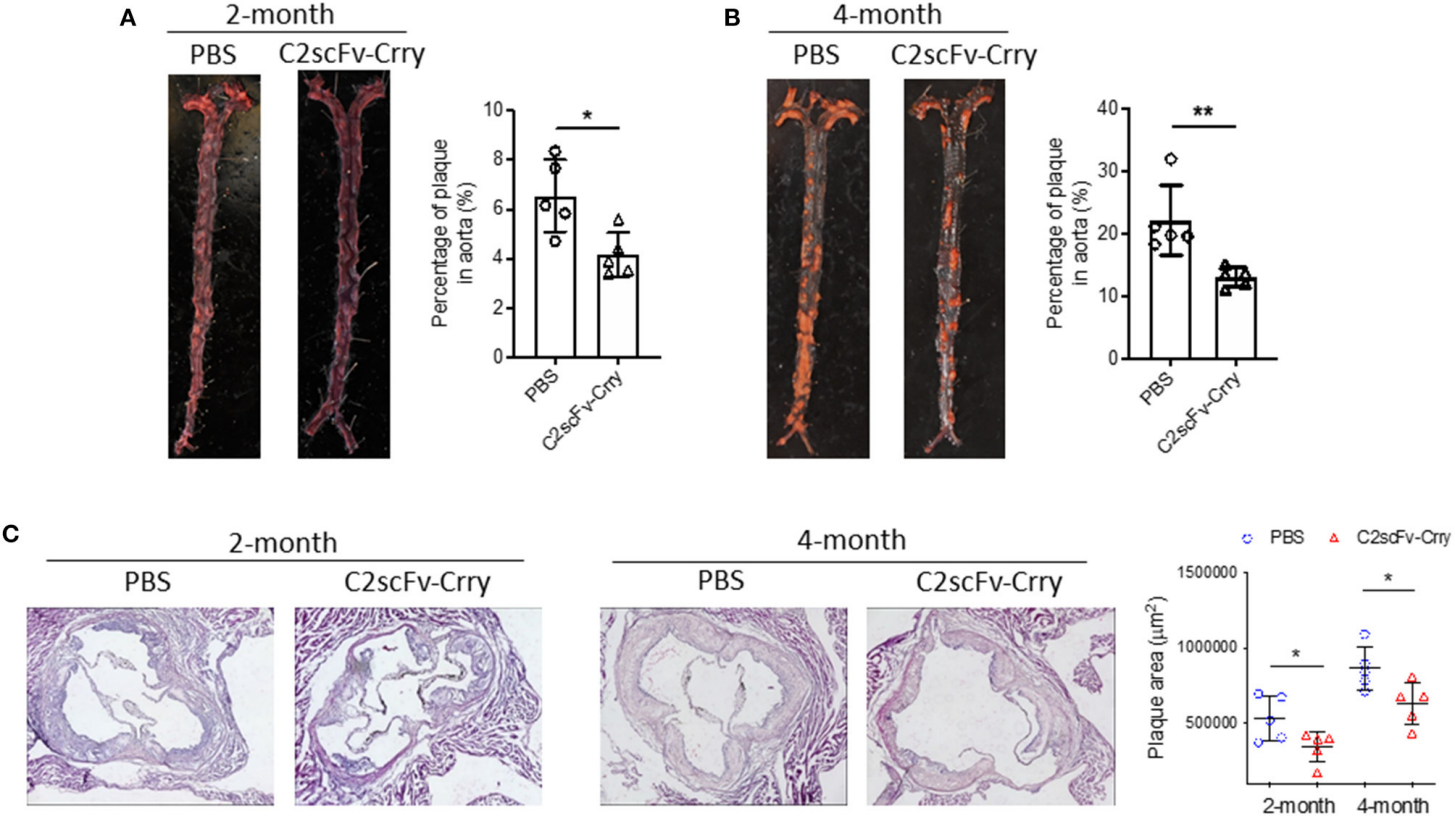
C2scFv-Crry attenuates atherosclerosis plaque in Apoe^−/−^ mice fed with high-fat diet (HFD) for 2 or 4 months. Apoe^−/−^ mice were fed a high-fat diet (HFD) and treated with PBS or C2scFv-Crry (0.25 mg/mice, twice a week) for 2 or 4 months, followed by quantification of atherosclerosis plaque. **(A,B)** Representative images (left) and quantification (right) of aortic en face plaque area determined by Oil Red O (ORO) staining for 2-month **(A)** and 4-month groups **(B)**. Quantification represents the percentage of ORO-stained plaque areas in the entire aorta. **(C)** H&E staining of cross-sections of aortic root in Apoe^−/−^ mice treated with PBS or C2scFv-Crry. Left: representative images. Right: quantification plaque aera in aortic root (^*^p < 0.05, ^**^p < 0.01 using unpaired Student's *t*-test).

To determine the therapeutic effect of C2scFv-Crry in the development of atherosclerosis, we treated 2-month-old *Apoe*^−/−^ mice with C2scFv-Crry (0.25 mg/mouse) or PBS (twice per week) for either 2 or 4 months and maintained the mice on a high-fat diet (HFD), following which we examined atherosclerotic plaque in the whole aorta and aortic root. *En face* Oil Red O (ORO) staining of the aorta showed that C2scFv-Crry-treated mice had significantly less plaque area on the aortic surface than PBS-treated mice after both 2 and 4 months of treatment (Figures 2A,B). Of note, although C2scFv-Crry treatment did not affect body weight at either time point, the weight of abdominal fat tissue was reduced in mice treated with C2scFv-Crry for 2 months as compared to PBS (Supplementary Figures 1A,B). Further, H&E staining of aortic root sections showed reduced plaque area in aortic roots of C2scFv-Crry-treated mice compared to PBS-treated mice at both time points (Figure 2C). Together, these results demonstrate that C2scFv-Crry treatment attenuates the development of atherosclerosis in mice.

### C2scFv-Crry-Treated Mice Have Significantly Less Complement Activation in the Plaque Than PBS-Treated Mice

**Figure 3:**
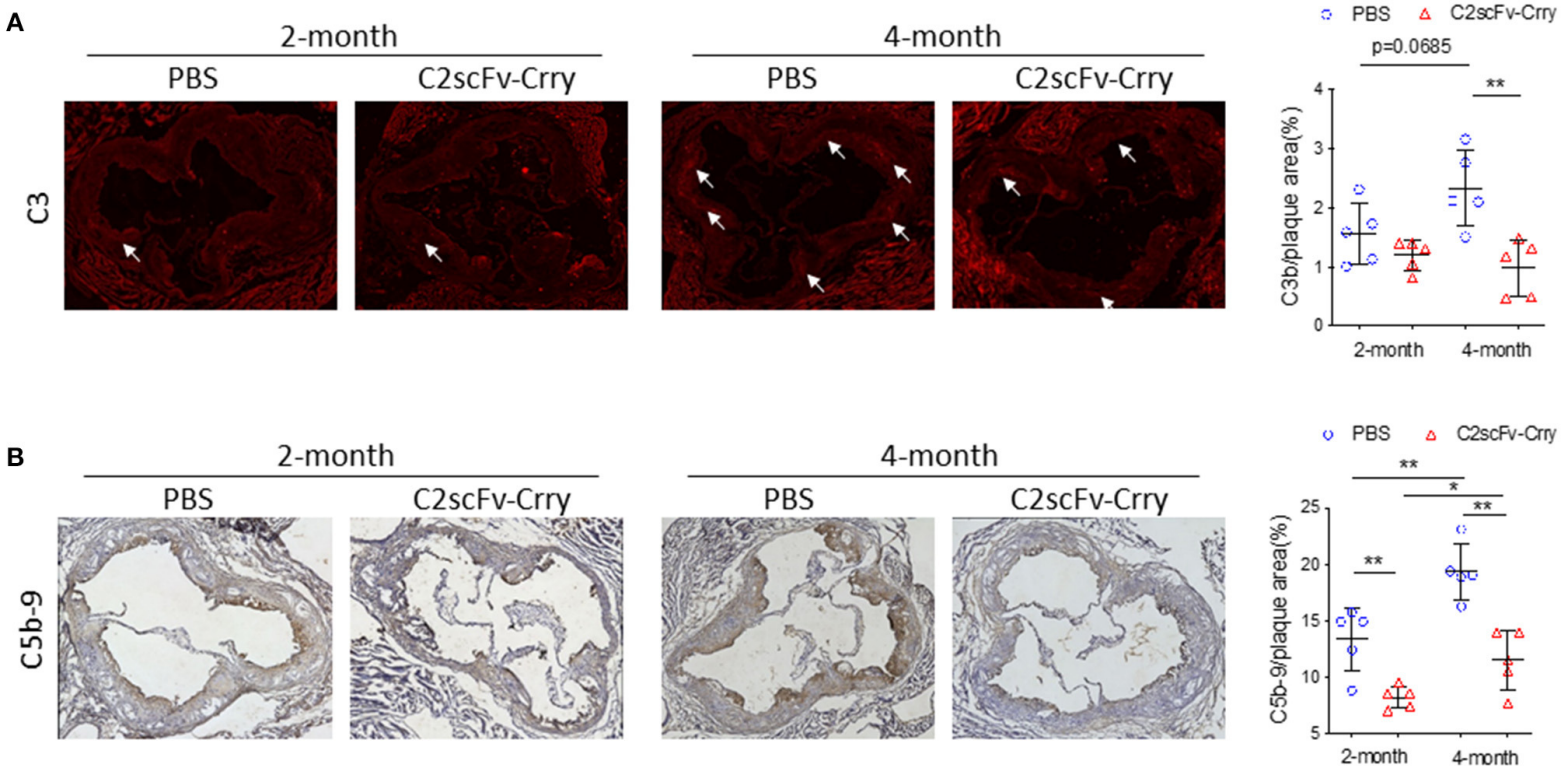
C2scFv-Crry attenuates complement activation in the plaque of Apoe^−/−^ mice. **(A)** Representative C3b (left) staining in the aortic root from Apoe^−/−^ mice treated with PBS or C2-Crry and quantification of C3b positive area in plaque (right). **(B)** Representative C5b-9 (left) staining in the aortic root from Apoe^−/−^ mice treated with PBS or C2-Crry and quantification of C5b-9 positive area in plaque (right) (^*^p < 0.05, ^**^p < 0.01 using unpaired Student's *t*-test).

To investigate whether the anti-atherogenic effect of C2scFv-Crry is associated with the targeted inhibition of the complement system, we first analyzed C3 activation in the plaque of these mice. Immunostaining using an antibody specific for deposited C3 activation products (C3b/iC3b/C3c) showed significantly less C3 deposition in aortic roots from *Apoe*^−/−^ mice treated with C2scFv-Crry for 4 months compared to PBS controls (Figure 3A). We previously demonstrated that the MAC has an atherogenic role. To this end, we further explored the functional connection between C2scFv-Crry treatment and MAC formation. Using an antibody to C5b-9/MAC, we found that C2scFv-Crry-treated mice had significantly less MAC deposition in the plaque of aortic roots than PBS-treated mice in both the 2- and 4-month groups (Figure 3B). To investigate whether the MAC may be inducing apoptosis, which may stimulate the formation of vulnerable plaque (51). We measured the apoptosis level in the plaque using TUNEL staining. We found that C2scFv-Crry-treated mice had very low, but similar levels of TUNEL positive area compared to PBS-treated mice in both the 2- and 4-month groups, suggesting C2scFv-Crry does not act through an effect from apoptosis in this model (Supplementary Figure 2). Furthermore, the area of CD68+ macrophage and SMA-α+ smooth muscle cell in the plaque was not changed by C2scFv-Crry (Supplementary Figure 3), suggesting that C2scFv-Crry may not function through affecting immune cell composition in the plaque. Altogether, these results demonstrate that the atheroprotective effect of C2scFv-Crry is associated with the targeted inhibition of complement activation in plaque, further supporting the therapeutic potential of complement inhibition in atherosclerosis.

### C2scFv-Crry Inhibits Oxidized LDL-Induced Inflammation *in vitro* and Lipid Deposition in Plaque

**Figure 4:**
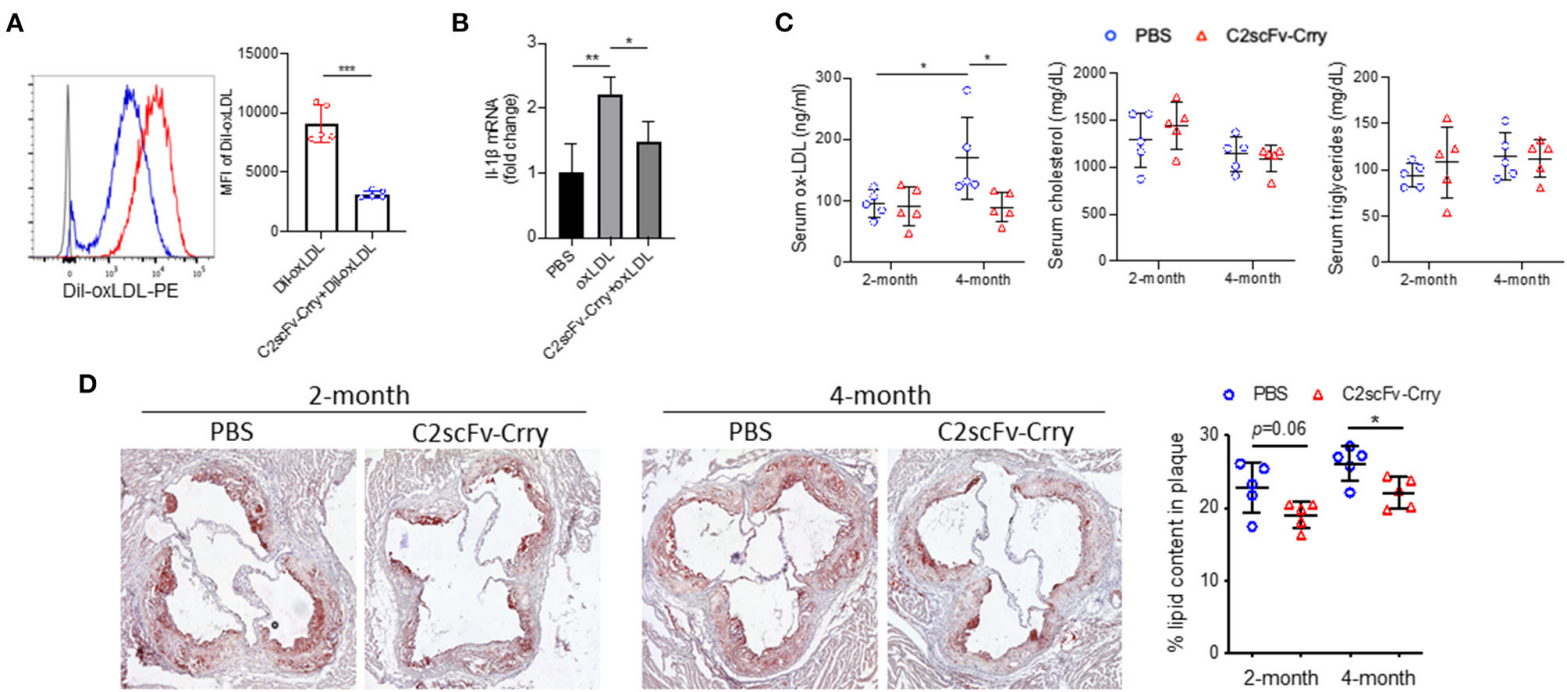
C2scFv-Crry inhibits oxidized low-density-lipoprotein uptake by macrophage and lipid deposition in the plaque. **(A)** Dil-labelled oxidized low-density lipoprotein (Dil-oxLDL) (50 ug/ml) was pre-incubated with PBS or 20 ug/ml C2scFv-Crry for 30 min, followed by incubation with peritoneal macrophage for 16 h. The uptake of Dil-oxLDL was determined by the intensity of Dil in macrophages. Left: Representative histogram of Dil. Right: Mean fluorescence intensity (MFI) of Dil. **(B)** IL-1ß mRNA level in peritoneal macrophage treated with pbs, oxLDL or oxLDL+C2scFv-Crry for 24 h. **(C)** Serum oxLDL, cholesterol and triglyceride levels in Apoe^−/−^ mice treated with PBS or C2scFv-Crry. **(D)** Representative image and quantification of Oil Red O staining in aortic root of Apoe^−/−^ mice treated with PBS or C2scFv-Crry (^*^p < 0.05, ^**^p < 0.01, ^***^p < 0.005 using unpaired Student's *t*-test).

Since the C2 mAb was originally characterized as an antibody that recognizes injury-exposed phospholipids normally recognized by natural circulating IgM, and phospholipids make up the outer shell of lipoproteins, we examined the effect of C2scFv-Crry on lipoprotein uptake, which is a critical event in the progression of atherosclerosis. To this end, we performed an oxLDL uptake assay in which peritoneal macrophages were incubated with fluorescence (Dil)-labeled oxLDL (Dil-oxLDL), followed by flow cytometry analysis of Dil fluorescence intensity in macrophages. We found that pre-incubation of C2scFv-Crry with Dil-oxLDL significantly inhibited the uptake of oxLDL by macrophages, as illustrated by reduced Dil intensity (Figure 4A). In addition, oxLDL has been demonstrated to promote inflammatory cytokine expression (26). We found that C2scFv-Crry counteracted oxLDL-induced IL-1β upregulation in macrophages (Figure 4B). *In vivo*, although C2scFv-Crry-treated mice had similar levels of serum cholesterol and triglycerides compared to PBS-treated mice, serum oxLDL levels were significantly reduced in mice treated with C2scFv-Crry for 4 months (Figure 4C), suggesting that long-term administration of C2scFv-Crry can lower the oxidization of LDL. Finally, ORO staining revealed that C2scFv-Crry-treated mice had significantly lower lipid content in aortic root plaques than PBS-treated mice at both time points (Figure 4D). Together, these data suggest that in addition to inhibiting complement activation, C2scFv-Crry also attenuates lipid deposition, oxidization, and uptake in plaques, possibly mediated by its C2 moiety.

### C2scFv-Crry Reduces Endogenous IgM Deposition in the Plaque

**Figure 5:**
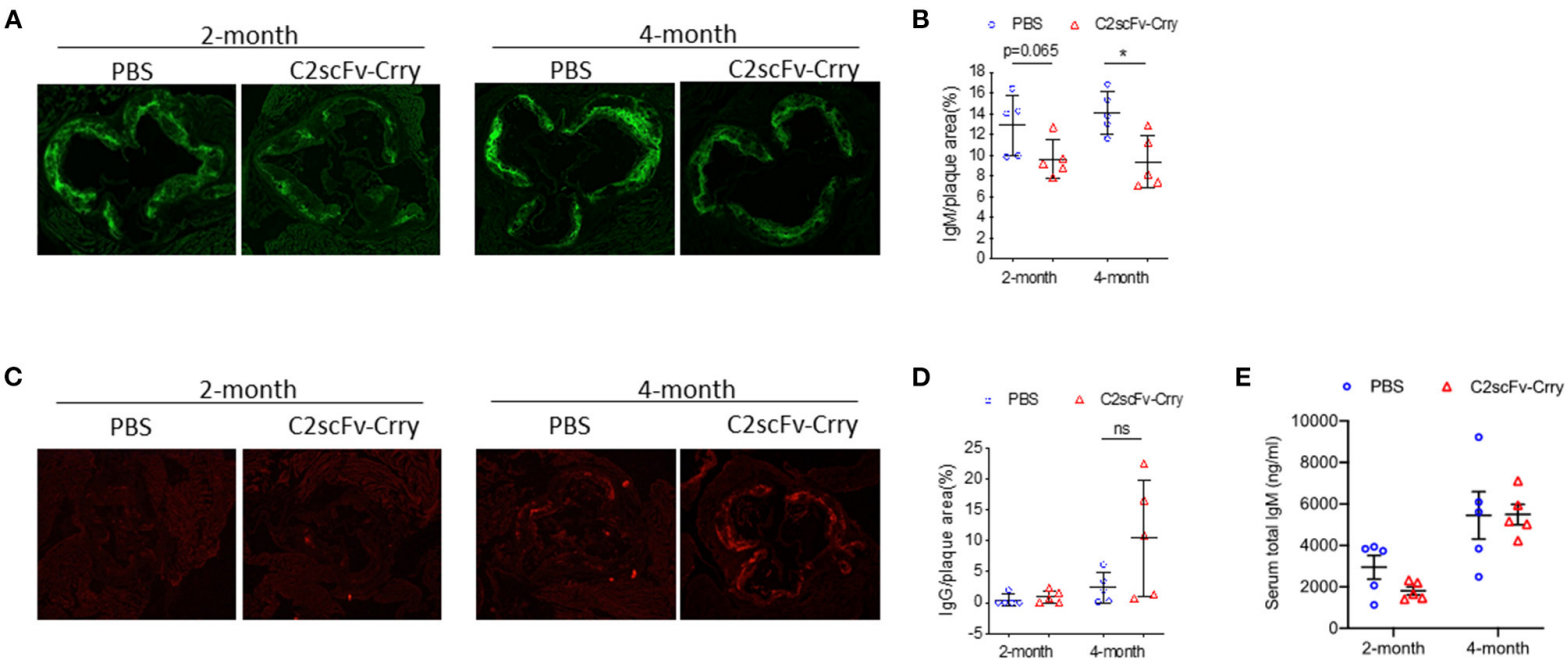
C2scFv-Crry specifically decreases IgM levels in the plaque but not the serum of Apoe^−/−^ mice. **(A)** Representative total IgM staining in the aortic root of Apoe^−/−^ mice treated with PBS or C2scFv-Crry. **(B)** Quantification of total IgM positive area in the plaque. **(C)** Representative total IgG staining in the aortic root of Apoe^−/−^ mice treated with PBS or C2scFv-Crry. **(D)** Quantification of total IgG positive area in the plaque. **(E)** Serum total IgM level in Apoe^−/−^ mice (^*^p < 0.05 using unpaired Student's *t*-test).

A previous study demonstrated that natural IgM recognizing oxidized phospholipids deposit in plaques and play a protective role in atherosclerosis (52). To determine whether the infusion of C2scFv-Crry has any effect on endogenous natural antibody deposition, we analyzed the level of total IgM and IgG in the plaque after C2scFv-Crry treatment. Using an antibody specific for total IgM or IgG, but not single-chain fragments, we found that mice treated with C2scFv-Crry for 2 and 4 months had reduced levels of endogenous IgM, but not IgG, in the aortic root plaque compared to mice treated with PBS (Figures 5A–D). This effect is plaque-specific, since IgM levels in serum is not changed (Figure 5E). These results indicate that C2scFv-Crry treatment impairs the deposition of endogenous phospholipid-specific IgM in the plaque, which may result from the competitive binding of phospholipids by the single-chain fragment of C2. The atheroprotective effect of C2scFv-Crry is thus likely due to the combined inhibition of complement by Crry and lipid uptake by single-chain of C2.

## Discussion

Here we report a novel complement inhibitory strategy to protect against the development of atherosclerosis. A potential mechanism for the protective role of C2scFv-Crry is localized inhibition of complement activation and reduced lipid deposition at site of pathology and the subsequent dampening of a sterile inflammatory response. The C2 IgM mAb, from which the targeting moiety was derived, was initially identified as binding to a subset of phospholipids displayed specifically on injured cells following cerebral ischemia. Here we demonstrate that similar phospholipid neoepitopes are expressed in atherosclerotic plaques, and that targeting a complement inhibitor to these neoepitopes is anti-atherogenic and reduces local complement activation as evidenced by decreased C3 deposition and MAC formation. The targeting of C2scFv-Crry was confirmed by immunostaining showing the localization of C2scFv-Crry in the plaque. Since the C2scFv targeting moiety recognizes neoepitopes exposed in the plaque, phospholipids, which are a typical component of plaques, represent epitopes that could be used for the targeted delivery of therapeutics to treat atherosclerosis. It is important to note that our current study mainly focuses on the effect of C2scFv-Crry, and the role of IgM and complement, in the development of the plaque, and as such does not represent a therapeutic examining the effect of C2scFv-Crry on establishment of the plaques. Thus, the therapeutic effect of C2scFv-Crry in established atherosclerosis remains unclear and requires further investigation.

In addition to complement inhibition, we also demonstrate that C2scFv-Crry inhibits oxLDL uptake *in vitro* and lipid content in the plaque. A critical event in the early stage of plaque development is the deposition and oxidization of LDL (53). OxLDL accumulates with the progression of plaque and contributes to plaque development (35, 36). A recent study showed that oxidized phospholipids, active components of oxLDL, are proinflammatory and proatherogenic in mice (46), suggesting the therapeutic potential of targeting oxLDL. Indeed, it has been recognized that natural IgM against oxidized phospholipids plays an anti-atherogenic role in the context of atherosclerosis (54–56). For example, the natural IgM antibody T15/E06, which was isolated from cholesterol-fed apoE mice and binds to oxidized phospholipids, blocks the uptake of oxLDL by macrophages and reduces vascular inflammation (57). In addition, passive infusion of T15/E06 reduced vein-graft atherosclerosis in *Apoe*^−/−^ mice (58). In our study, we found that C2scFv-Crry significantly attenuated oxLDL uptake and inflammation, and reduced lipid content in plaques, suggesting that in addition to complement inhibition mediated by Crry, C2scFv-Crry may also act on lipid deposition in the plaque through its C2scFv component. Interestingly, we also found that 4 months (but not 2 months) of C2scFv-Crry treatment reduced serum oxLDL levels, an indicator of LDL oxidization in plaques (59). These results indicate that long-term C2scFv-Crry treatment inhibits the oxidization of LDL, which may further contribute to its atheroprotective effect. However, further study is required to determine the exact mechanism.

It is known that natural IgM recognizing neoepitopes on stressed and injured cells can activate complement and drive pathology in various disease conditions. Although a previous study has demonstrated an overall beneficial role of lipid-targeting natural IgM in atherosclerosis, the role of IgM in complement activation has not been determined in atherosclerosis. Whereas the C2 mAb can induce complement activation via an Fc fragment, as demonstrated in a lung transplant injury model (45), the C2scFv-Crry construct does not contain an Fc fragment and thus lacks the ability to activate complement. An area for future study will be the role of natural antibody in complement activation in driving atherosclerosis. In this context, we observed decreased deposition of IgM in the plaque following C2scFv-Crry treatment. A potential explanation for this is competitive binding of C2scFv-Crry to oxLDL in the plaque, which attenuates the deposition of endogenous IgM. It will be interesting to investigate the therapeutic effect of other oxidized phospholipid-targeted IgM-mediated complement inhibitors in atherosclerosis, and which may have additive effects by blocking IgM binding to multiple epitopes. Interestingly, C2scFv-Crry treatment does not change the level of total IgG in the plaque, suggesting IgG may not involve in complement activation in the current model. Furthermore, we did not observe any change in macrophage and SMC content in the plaque. It has been shown that oxLDL uptake induces macrophage polarization toward alternative M2-like types, which take up a higher level of oxLDL than M1-like macrophages *in vitro* (60, 61). The dynamic change of macrophage phenotype and activation status *in vivo* after C2scFv-Crry treatment and whether there are other cell types involved requires further investigation.

In summary, we have demonstrated that C2scFv-Crry protects mice against atherosclerosis by targeting inhibition of complement activation and pro-inflammatory oxLDL uptake and deposition, which represents a novel targeted approach of complement inhibition for the treatment of atherosclerosis.

## Data Availability Statement

The original contributions presented in the study are included in the article/Supplementary Material, further inquiries can be directed to the corresponding author.

## Ethics Statement

The animal study was reviewed and approved by Institutional Animal Care and Use Committee (IACUC) of Tulane University and Temple University.

## Author Contributions

SD, FL, X-FY, HW, ST, and XQ developed the concept. SD, FL, MR, ZQ, NR, and XQ contributed to perform the experiments and analyze the results. ST provided the complement inhibitors. SD, FL, NR, X-FY, HW, ST, and XQ wrote the manuscript, and all authors participated in the review and critique of the manuscript. HW, ST, and XQ interpreted the results and supervised the experiments.

## Funding

This work was supported by R21OD024931 (XQ), R01 HL130233 (HW and XQ), and by grants from the VA, BX 005235, BX004256, RX001141, and the DOD W81XWH2010743 (ST).

## Conflict of Interest

ST is a consultant for Q32 Bio, a company developing complement inhibitors, and is an inventor on a licensed patent for targeting constructs based on natural antibody. ST is a co-founder of Q32 Bio and owns equity in the company. The remaining authors declare that the research was conducted in the absence of any commercial or financial relationships that could be construed as a potential conflict of interest.

## Publisher's Note

All claims expressed in this article are solely those of the authors and do not necessarily represent those of their affiliated organizations, or those of the publisher, the editors and the reviewers. Any product that may be evaluated in this article, or claim that may be made by its manufacturer, is not guaranteed or endorsed by the publisher.

## Abbreviations

scFv: Single chain fragment variable
HFD: High-fat diet
MAC: Membrane attack complex
oxLDL: Oxidized low-density lipoprotein
hCD59: Human CD59
ORO staining: Oil-red-O staining
SMC: Smooth muscle cell
SMA-α: Alpha smooth muscle actin
TUNEL: Terminal deoxynucleotidyl transferase dUTP nick end labeling.
