# Supplementary material for: Complement Inhibition Targeted to Injury Specific Neoepitopes Attenuates Atherogenesis in Mice
Source: Front Cardiovasc Med. 2021 Sep 28;8:731315. doi: 10.3389/fcvm.2021.731315 (PMC8505745; doi:10.3389/fcvm.2021.731315)
Supplement: Supplementary file 1 [file Data_Sheet_1.PDF]

Supplemental Figure 1

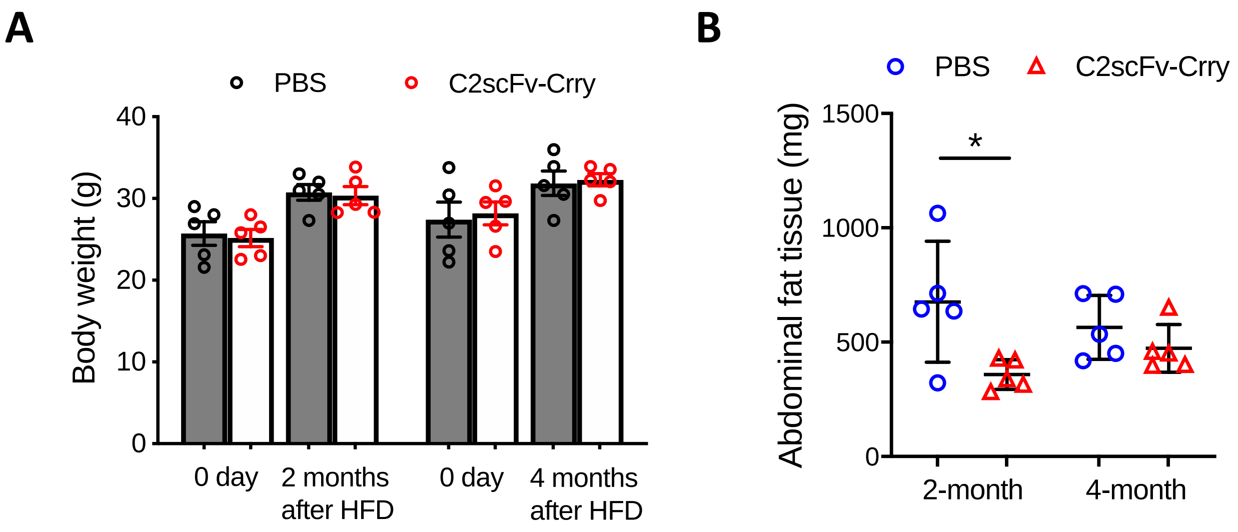

Supplemental Figure 1. The effect of C2scFv-Crry in the body weight and abdominal fat tissue in *Apoe*$^{-/-}$ mice.

Supplemental Figure 2

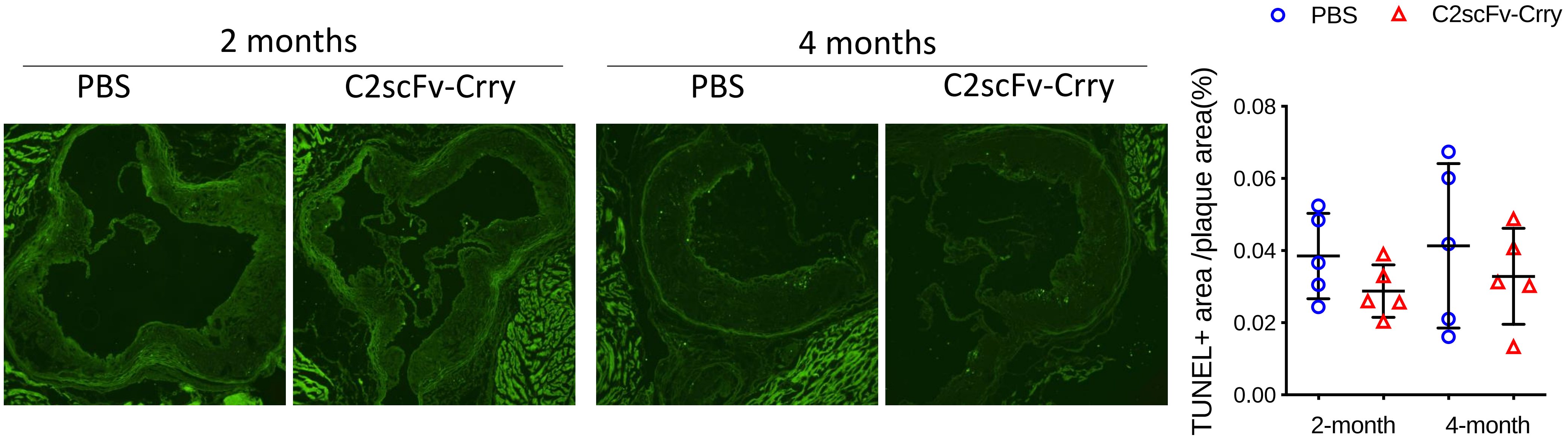

Supplemental Figure 2. C2scFv-Crry dose not affect apoptosis in the plaque of *Apoe*$^{-/-}$ mice. Representative TUNEL staining (left) in the aortic root of *Apoe*$^{-/-}$ mice treated with PBS or C2scFv-Crry and the quantification of TUNEL positive area in plaque (right).

Supplemental Figure 3

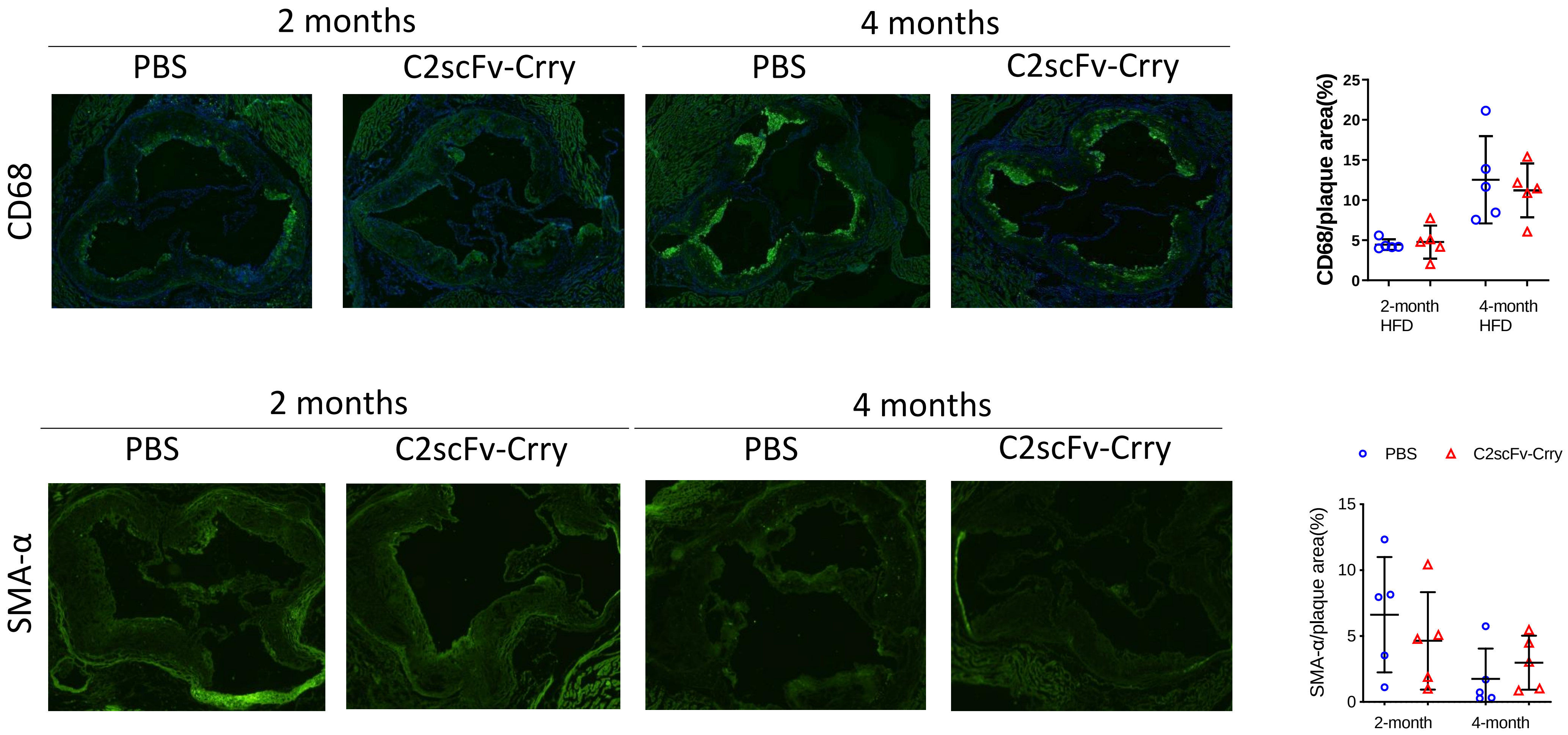

Supplemental Figure 3. C2scFv-Crry dose not change the content of macrophage and SMC in the plaque of *Apoe*$^{-/-}$ mice. Representative CD68 (upper, left) and SMA-α (lower, left) staining in the aortic root of *Apoe*$^{-/-}$ mice treated with PBS or C2scFv-Crry and the quantification of CD68/ SMA-α positive area in plaque (right).
